# Army Medical School

**Published:** 1860-07

**Authors:** 


ARMY MEDICAL SCHOOL.—The Royal Sanitary Commission has within the past three years produced many great reforms in the Medical Department of the British army, the last being the constitution of the New Army Medical School, at Chatham, as a distinct and independent institution. Its course of instruction will not supersede that at the different English Medical Schools, but will be of a practical nature, and devoted exclusively to the requirements of the military medical service. The senate of the school, in which is vested its government, is constituted of the following gentlemen:—Dr. T. B. Gibson, Director-General; J. R. Martin, Esq., Physician to the Council of India; J. R. Taylor, C.B., Inspector-General of Hospitals, and Principal Medical Officer at Fort Pitt, Chatham; T. Longmore, Esq., Deputy Inspector-General and Professor of Clinical and Military Surgery; C. Morehead, M.D., Professor of Clinical and Military Medicine; E. A. Parkes, M.D., Professor of Hygiene; W. Aitken, M.D., Professor of Pathology.

These appointments will doubtless give universal satisfaction to the medical profession of Great Britain, as all the professors are men of high reputation. The buildings are erected at Fort Pitt Hospital, and the first course of lectures will commence after the next examination for the Army Medical and India Medical Services.

